# Supplementary material for: miRNA Expression Is Increased in Serum from Patients with Semantic Variant Primary Progressive Aphasia
Source: Int J Mol Sci. 2022 Jul 30;23(15):8487. doi: 10.3390/ijms23158487 (PMC9368911; doi:10.3390/ijms23158487)
Supplement: Supplementary file 1 [file ijms-23-08487-s001.zip › ijms-1819593-supplementary.pdf]

Supplementary Table S1: List of 479 mRNA target overlapped between miR-133a-3p and miR-106b-5p using two bioinformatic tools: TargetScan and miRDB.

LASP1
BCL2L2
TRIQK
GDNF
TAGLN2
CORO1C
WASF2
SYT2
PTBP2
GABARAPL1
GPM6A
SCN2B
DUSP1
DCLRE1A
RAP2C
STOM
NRIP3
SHISA5
FOXL2
AKAP9
ZNF280C
RCL1
THRAP3
VAPB
CTBP2
STX5
CERS2
LHX5
PFKFB3
MECOM
ZNF436
SLC7A8
VAT1
SOGA1
PCDHGA12
FGF1
KCTD20
ARFIP2
CAPN15
NAGS
PML
YES1
MSN
CCDC117

FBXL2
PRRT2
SMARCD1
PRRX1
TFE3
GRM5
EMP2
FAM117B
HAPLN1
YPEL2
MAML3
GNAI3
TXLNA
TTYH3
POU4F1
SEC61B
KPNA6
PREX1
CAMKK2
PROX2
FAM49A
CNTNAP1
TMEM167A
KLHDC10
CACUL1
CELF5
PITPNM2
NELFA
FTL
GPR173
ZNF322
CETN3
MYH9
TFAP2D
PTH1R
CMTM6
KLF7
CDYL2
CLTA
TGFBR1
NIPA1
PAX7
TIMM17A
QKI
SF3B4
SLC50A1

PTBP1
PPP2CA
KLHL11
BICC1
CNN2
KIF3C
SIMC1
MLLT3
TBPL1
DOLPP1
VPS54
ATP6AP2
TRAM2
RARB
SNX15
SGMS2
SLC6A1
SFXN5
USP6
UNC13A
SGPP1
SLC30A7
ZC3H7A
SGK1
LPGAT1
MYRF
ZC3H11A
SV2A
SP3
AFTPH
TMOD3
LANCL2
CMPK1
ZNF131
RBMX
UBA2
PPP2CB
ELFN1
MRC2
ARHGAP12
ANKRD28
STXBP6
SOX4
FOXC1
RAVER1
CRTAM

GABPB2
PPFIA2
PLEKHA3
SYT1
PTPRZ1
RPL17-
C18orf32
LRRC7
NDRG1
UBE2Q1
PTPRK
PFN2
SESN1
SNRK
GLRA2
BCORL1
MEIS2
BTBD3
TMEM170B
ANKRD44
CDK13
EDEM1
RBPJ
EXD2
SLC6A6
SOBP
CPNE3
PAN3
XPO4
HS2ST1
SUPT16H
RBMXL1
GDI2
ARHGDIA
RIMS1
NIPA2
GXYLT1
PTPRD
ARHGAP9
SACM1L
MAML1
CREB5
RB1CC1
CRK
SGTB
MEIS1

DOT1L
FGFR1
GRID2
ANKRD12
FOXP4
MMP15
MED12L
SYNPO2L
EPHA7
UBXN7
ELAVL1
TENM1
PRDM16
GNB4
PEX5L
EPS15
PTBP3
AFAP1
SOGA3
FLVCR1
MLXIP
USP32
RUNX1T1
CSNK1G3
PIK3C2A
ASH1L
FAM160B1
TRHDE
SLC4A8
AMD1
CNOT1
NFAT5
ARRB1
JAZF1
CYLD
HIC2
RTN4RL1
WIPI2
FOXP2
CHD3
ADCYAP1
NUP153
ZC3H14
SRPK1
MCL1
GDAP2

ERP44
ZNF367
ARID4B
DYNC1LI2
SLITRK3
STAT3
NABP1
NR4A3
AKTIP
SMOC1
NPAS2
PPP1R21
USP3
AMER2
REEP3
KIF5A
CADM2
ARHGEF28
RBBP7
LDLR
FASTK
LAMP5
TGFB1I1
B3GALT2
ANKRD50
LAPTM4A
GNS
EREG
ARHGAP1
ZBTB4
BTBD7
UXS1
NAA30
FAM19A1
JAK1
NRSN1
S1PR1
NEUROG1
RAB12
HMBOX1
IRF1
CHD9
ERI1
YOD1
TNFSF11
NEDD4L

MAT2B
NEUROG2
TNKS1BP1
ZNF217
TMUB2
IQSEC1
PRDM6
PITPNA
UNKL
SLC41A1
TIMP2
ESR1
MYCN
FAM102A
ATE1
YPEL4
METAP1
PSD
RAPGEF4
TBC1D8B
LRRC55
MAP3K8
ARL4C
FRMD4A
ZNF704
PPP1R15B
MAP3K9
TNFAIP1
CABLES1
RNF38
ERBB3
RAPGEFL1
DAZAP2
PDGFRA
GBF1
NRBP1
ANKFY1
DCUN1D3
MYO1D
TMEM123
SUMF1
C2orf69
TSHZ3
NOL4
KLHL20
CRIM1

PAF1
FAM126B
ELK4
ZHX2
ARHGEF3
DAB2
IL25
POLQ
PAFAH1B2
SCAMP2
STX6
MAP7
PHF1
FRMD4B
RBMS1
HMGB3
PCDHA2
PCDHA10
PCDHA4
PCDHA7
PCDHA5
PCDHA12
WEE1
PCDHA11
PCDHAC1
PCDHA9
PCDHA6
PCDHA1
PCDHA13
PCDHA3
PCDHA8
CHIC1
PGBD5
MAP3K12
ZNF385A
PCDHAC2
KIAA1522
ARAP2
RPS6KA1
SCN2A
ZNF202
RND3
SUCO
NEK9
SMAD5
E2F2

SNTB2
BNC2
RNF145
PLEKHO2
PANK3
PLEKHM1
DIP2A
TLE4
CPEB3
FAM117A
SH3BP5
PKN2
SFMBT1
FXR1
TMEM168
YTHDF3
FGF4
KIF3B
EIF5A2
ITCH
BICD2
OCRL
BBX
SLC35F3
NEURL1B
SNX21
MAPK4
CEP170
EPHB4
DCBLD2
RACGAP1
SKI
HECA
STYX
NFIB
RAB30
MAP3K1
SLC4A4
VEGFA
DEDD
CHD5
PLCB1
CIT
PPP6R3
NDEL1
GOSR1

LIMK1
RGS4
CYP26B1
AKT3
SALL1
TBX3
PHIP
STK11IP
RASGEF1A
ACSL4
ZNF532
MYNN
MCM3
IL1RAP
INO80
FOXA1
ZNF236
KIF26B
FNDC3A
C16orf72
RPS6KA2
SRGAP1
IPO9
SLC24A4
IPCEF1
THBS2
UBR5
KMT2C
TBC1D2
COL4A1
ALX4
DUSP8
TSKU
SULF1
FMNL3
CREB1
MYCL
FGF5
ANKRD29
UBASH3B
RAB11FIP4
TSPAN9
ACBD5
IGSF10
SSH1
AFF4

SLC25A36
LRP1B
ZBTB6
ABI1
CELSR2
SP8
ITFG1
ZRANB1
HP1BP3
UBE2Q2
SPTY2D1
ZNF652
GID4
TMEM50B
MECP2
SRGAP3
SESN3
NUFIP2
RPS6KA6
GGCX
PPP6R2
UBASH3A

Supplementary Table S2: gene target involved in cerebral physiopathology

Overlapping target mRNAs between miR-133a-3p and miR-106b-5p	Involvement in neurological conditions or neurobiology	References
ARHGAP12	Synapse development	Ba et al 2016
CMPK1		
CRK		
DERL2	Brain atrophy in the cerebellum and striatum, reduced neurite outgrowth and motor function in KO mice	Sugiyama et al, 2021
DNAJB6	Anti-amyloidogenic in neurons	Thiruvalluvan et al, 2020
DYNC1LI2	Motor and sensory neuron loss in mice with dynein mutation	Deng et al, 2010
ENPP5	upregulated in astrocytes, oligodendrocytes and neurons of AD mouse model	Pietrowski et al, 2021
EPHA7	Neurodevelopment disorder	Lévy et al, 2021
FAM117B	Associated with genetic risk of AD	Madrid et al, 2021
FAM13A		
FBXW11	Neuroprotection, increased espression in AD mouse model	Sun et al, 2021
FOXL2		
FOXQ1	Senescence, inflammation/ AD cellular model	Wang et al, 2017<br>Zhuang et al, 2020<br>Maiese, 2016
FURIN	Neuronal cell death	Yamada et al, 2018
HLF	Epilepsy, mouse model	Hawkins & Kearny, 2018
HMBOX1	Associated with genetic risk of AD	Li et al, 2021
HS3ST5	Associated with genetic risk of AD	Bellenguez et al, 2022<br>Pérez-López et al, 2021
KLHL2		
LASP1	Associated with global cognitive function, category verbal fluency, and spatial working memory of patients with schizophrenia	Li et al, 2019
LPGAT1	AD (cell model)	Lin et al, 2018
MAP3K2		
MED12L	Intellectual disability developmental delay or speech impairment in individuals with MED12L deletion	Nizon et al, 2019
MINK1	Synapse loss in AD	Lawingco et al, 2021
NTNG1	Cortical layer structure in mammals	Yaguchi et al, 2014
PFN2	Associated with Charcot-Marie-Tooth type 2	Juneja et al, 2018
PLEKHA3		
PRDM6		
RAP2C		
RRAGD		
SGTB		

SLC41A1	Associated with genetic risk for PD	Li et al, 2021, Cibulka et al, 2022
SMIM14	FTD	Dickson et al, 2019
SOBP		
SOX4		
TIMM17A	Mithocondrial protein downregulated in AD	Liang et al, 2008 Bogorodskiy et al, 2021
TMEM64		
TTPAL	Differential expressed in AD	Li et al, 2015
YPEL2		
ZNF385A		

ARHGAP12, Rho GTPase Activating Protein 12; CMPK1, Cytidine/Uridine Monophosphate Kinase; CRK, CRK Proto-Oncogene, Adaptor Protein; DNAJB6, DnaJ Heat Shock Protein Family (Hsp40) Member B6; DYNC1LI2, motorDynein Cytoplasmic 1 Light Intermediate Chain 2; ENPP5, Ectonucleotide Pyrophosphatase/Phosphodiesterase Family Member 5; EPHA7, ephrin receptor A7; FAM117B, Family With Sequence Similarity 117 Member B; FAM13A, Family With Sequence Similarity 13 Member A; FBXW11, F-box and WD-40 domain protein 11; FOXL2, forkhead box L2; FOXQ1, Forkhead Box Q1; HLF, HLF transcription factor; HMBOX1, Homeobox Containing 1; HS3ST5, Heparan Sulfate-Glucosamine 3-Sulfotransferase 5; KLHL2, Kelch-like protein family member 2; LASP1, LIM And SH3 Protein 1; LPGAT1, lysophosphatidylglycerol acyltransferase 1; netrin-G1, Mediator Complex Subunit 12L, MED12L; MAP3K2, Mitogen-activated Protein Kinase Kinase Kinase 2; MINK1, Misshapen Like Kinase 1;; NTNG1, Netrin-G1; PFN2, Profilin-2; PLEKHA3, Pleckstrin Homology Domain-Containing Family A Member 3; PRDM6, Putative histone-lysine N-methyltransferase; RAP2C, Ras-Related Protein Rap-2c; RRAGD, Ras-Related GTP-Binding Protein D; SGTB, , Small Glutamine Rich Tetratricopeptide Repeat Co-Chaperone Beta, SLC41A1, Solute Carrier Family 41 Member 1; SMIM14 Small Integral Membrane Protein 14; SOBP, Sine Oculis Binding Protein Homolog; SOX4, SRY-Box Transcription Factor 4; TIMM17A, Translocase Of Inner Mitochondrial Membrane 17A; TMEM64Transmembrane Protein 64; TTPAL, alpha tocopherol transfer protein like; YPEL, yippee like; ZNF385A, Zinc Finger Protein 385A.

References not included in the main text

Wenhan Deng, Caroline Garrett, Benjamin Dombert, Violetta Soura, Gareth Banks, Elizabeth M.C. Fisher, Marcel P. van der Brug, Majid Hafezparast,
Neurodegenerative Mutation in Cytoplasmic Dynein Alters Its Organization and Dynein-Dynactin and Dynein-Kinesin Interactions*, Journal of Biological Chemistry, Volume 285, Issue 51, 2010, Pages 39922-39934, ISSN 0021-9258, https://doi.org/10.1074/jbc.M110.178087

Pietrowski Marie J., Gabr Amr Ahmed, Kozlov Stanislav, Blum David, Halle Annett, Carvalho Kevin. Glial Purinergic Signaling in Neurodegeneration. Frontiers in Neurology, 12, 2021,
https://www.frontiersin.org/article/10.3389/fneur.2021.654850
DOI=10.3389/fneur.2021.654850

Madrid, L., Moreno-Grau, S., Ahmad, S., González-Pérez, A., de Rojas, I., Xia, R., Martino Adami, P. V., García-González, P., Kleineidam, L., Yang, Q., Damotte, V., Bis, J. C., Noguera-Perea, F., Bellenguez, C., Jian, X., Marín-Muñoz, J., Grenier-Boley, B., Orellana, A., Ikram, M. A., Amouyel, P., … Saez, M. E. (2021). Multiomics integrative analysis identifies APOE allele-specific blood biomarkers associated to Alzheimer's disease etiopathogenesis. Aging, 13(7), 9277–9329. Advance online publication. https://doi.org/10.18632/aging.202950

Sun J, Qin X, Zhang X, Wang Q, Zhang W, Wang M. FBXW11 deletion alleviates Alzheimer's disease by reducing neuroinflammation and amyloid-β plaque formation via repression of ASK1 signaling. Biochem Biophys Res Commun. 2021 Apr 9;548:104-111. doi: 10.1016/j.bbrc.2020.12.081. Epub 2021 Feb 25. PMID: 33640602.

Maiese K. (2016). Forkhead transcription factors: new considerations for alzheimer's disease and dementia. Journal of translational science, 2(4), 241–247. https://doi.org/10.15761/JTS.1000146

Yamada, M., Hayashi, H., Yuuki, M. et al. Furin inhibitor protects against neuronal cell death induced by activated NMDA receptors. Sci Rep 8, 5212 (2018). https://doi.org/10.1038/s41598-018-23567-0

Li HD, Funk CC, McFarland K, Dammer EB, Allen M, Carrasquillo MM, Levites Y, Chakrabarty P, Burgess JD, Wang X, Dickson D, Seyfried NT, Duong DM, Lah JJ, Younkin SG, Levey AI, Omenn GS, Ertekin-Taner N, Golde TE, Price ND. Integrative functional genomic analysis of intron retention in human and mouse brain with Alzheimer's disease. Alzheimers Dement. 2021 Jun;17(6):984-1004. doi: 10.1002/alz.12254.

Lawingco T, Chaudhury S, Brookes KJ, Guetta-Baranes T, Guerreiro R, Bras J, Hardy J, Francis P, Thomas A, Belbin O, Morgan K. Genetic variants in glutamate-, Aβ-, and tau-related pathways determine polygenic risk for Alzheimer's disease. Neurobiol Aging. 2021 May;101:299.e13-299.e21. doi: 10.1016/j.neurobiolaging.2020.11.009.

Zhuang Jingcong, Chen Zhongjie, Cai Pingping, Wang Rong, Yang Qingwei, Li Longling, Yang Huili, Zhu Renjing. Targeting MicroRNA-125b Promotes Neurite Outgrowth but Represses Cell Apoptosis and Inflammation via Blocking PTGS2 and CDK5 in a FOXQ1-Dependent Way in Alzheimer Disease. Frontiers in Cellular Neuroscience,14, 2020
https://www.frontiersin.org/article/10.3389/fncel.2020.587747
DOI=10.3389/fncel.2020.587747

Wang, P., Lv, C., Zhang, T. et al. FOXQ1 regulates senescence-associated inflammation via activation of SIRT1 expression. Cell Death Dis 8, e2946 (2017). https://doi.org/10.1038/cddis.2017.340

Bellenguez, C., Küçükali, F., Jansen, I.E. et al. New insights into the genetic etiology of Alzheimer’s disease and related dementias. *Nat Genet* **54,** 412–436 (2022). https://doi.org/10.1038/s41588-022-01024-z

Nizon, M., Laugel, V., Flanigan, K.M. et al. Variants in *MED12L*, encoding a subunit of the mediator kinase module, are responsible for intellectual disability associated with transcriptional defect. *Genet Med* **21,** 2713–2722 (2019). https://doi.org/10.1038/s41436-019-0557-3

Lawingco T, Chaudhury S, Brookes KJ, Guetta-Baranes T, Guerreiro R, Bras J, Hardy J, Francis P, Thomas A, Belbin O, Morgan K. Genetic variants in glutamate-, Aβ-, and tau-related pathways determine polygenic risk for Alzheimer's disease. Neurobiol Aging. 2021 May;101:299.e13-299.e21. doi: 10.1016/j.neurobiolaging.2020.11.009.

Yaguchi K, Nishimura-Akiyoshi S, Kuroki S, Onodera T, Itohara S. Identification of transcriptional regulatory elements for Ntng1 and Ntng2 genes in mice. Mol Brain. 2014 Mar 19;7:19. doi: 10.1186/1756-6606-7-19. PMID: 24642214; PMCID: PMC4000137.

Manisha Juneja, Abdelkrim Azmi, Jonathan Baets, Andreas Roos, Matthew J Jennings, Paola Saveri, Chiara Pisciotta, Nathalie Bernard-Marissal, Bernard L Schneider, Catherine Verfaillie, Roman Chrast, Pavel Seeman, Angelika F Hahn, Peter de Jonghe, Stuart Maudsley, Rita Horvath, Davide Pareyson, Vincent Timmerman. PFN2 and GAMT as common molecular determinants of axonal Charcot-Marie-Tooth disease
Journal of Neurology, Neurosurgery & Pyschiatry, 2018

Liang, W. S., Reiman, E. M., Valla, J., Dunckley, T., Beach, T. G., Grover, A., Niedzielko, T. L., Schneider, L. E., Mastroeni, D., Caselli, R., Kukull, W., Morris, J. C., Hulette, C. M., Schmechel, D., Rogers, J., & Stephan, D. A. (2008). Alzheimer's disease is associated with reduced expression of energy metabolism genes in posterior cingulate neurons. *Proceedings of the National Academy of Sciences of the United States of America*, *105*(11), 4441–4446. https://doi.org/10.1073/pnas.0709259105

Bogorodskiy, A., Okhrimenko, I., Burkatovskii, D., Jakobs, P., Maslov, I., Gordeliy, V., Dencher, N. A., Gensch, T., Voos, W., Altschmied, J., Haendeler, J., & Borshchevskiy, V. (2021). Role of Mitochondrial Protein Import in Age-Related Neurodegenerative and Cardiovascular Diseases. *Cells*, *10*(12), 3528. https://doi.org/10.3390/cells10123528

Li, X., Long, J., He, T. et al. Integrated genomic approaches identify major pathways and upstream regulators in late onset Alzheimer’s disease. *Sci Rep* **5,** 12393 (2015). https://doi.org/10.1038/srep12393

Pérez-López, Martín, C., García, B., Solís-Hernández, M. P., Rodríguez, D., Alcalde, I., Merayo, J., Fernández-Vega, I., & Quirós, L. M. (2021). Alterations in the Expression of the Genes Responsible for the Synthesis of Heparan Sulfate in Brains With Alzheimer Disease. Journal of Neuropathology and Experimental Neurology, 80(5), 446–456. https://doi.org/10.1093/jnen/nlab028

Pérez-López, Martín, C., García, B., Solís-Hernández, M. P., Rodríguez, D., Alcalde, I., Merayo, J., Fernández-Vega, I., & Quirós, L. M. (2021). Alterations in the Expression of the Genes Responsible for the Synthesis of Heparan Sulfate in Brains With Alzheimer Disease. *Journal of Neuropathology and Experimental Neurology*, *80*(5), 446–456. https://doi.org/10.1093/jnen/nlab028

Lin, Seo, J., Gao, F., Feldman, H. M., Wen, H.-L., Penney, J., Cam, H. P., Gjoneska, E., Raja, W. K., Cheng, J., Rueda, R., Kritskiy, O., Abdurrob, F., Peng, Z., Milo, B., Yu, C. J., Elmsaouri, S., Dey, D., Ko, T., … Tsai, L.-H. (2018). APOE4 Causes Widespread

Molecular and Cellular Alterations Associated with Alzheimer’s Disease Phenotypes in Human iPSC-Derived Brain Cell Types. Neuron (C
ambridge, Mass.), 98(6), 1294–1294. https://doi.org/10.1016/j.neuron.2018.06.011
